# The usefulness of head computed tomography in patients with known cirrhosis presenting to emergency department with suspected hepatic encephalopathy

**DOI:** 10.1093/jcag/gwae022

**Published:** 2024-07-10

**Authors:** David Yi Yang, Joel Bowron, Mohammed Ahmed, Juan G Abraldes, Sander Veldhuyzen van Zanten

## Abstract

**Background:**

Computed tomography of the head (CT head) is frequently used for patients with cirrhosis presenting with suspected hepatic encephalopathy (HE).

**Aims:**

The primary aims of this study were to assess the frequency of CT head usage in this patient population and to determine whether these scans yielded significant findings. Our secondary aims were to identify factors associated with the decision to order CTs and whether patients who received CTs had different outcomes.

**Methods:**

A single-centre, retrospective chart review was performed. Patients presenting to the University of Alberta Hospital with cirrhosis and common liver disease aetiologies over a 27-month period were identified via discharge diagnosis codes. Charts of patients with suspected HE were manually identified. The use of a CT head was documented, as were patient demographics, cirrhosis aetiology, MELD, and outcomes. Comparisons were made between patients with and without CT head.

**Results:**

A total of 119 encounters from 100 patients met our inclusion criteria. In 57% of encounters, a CT scan was performed on presentation. None of these CT scans had significant findings. Patient factors associated with the decision to order CT included older age, more preserved liver function, and longer length of time between patient’s current and previous presentations. Patients who did not receive CT head had higher in-hospital mortality, which was likely reflective of more severe underlying liver dysfunction in this group.

**Conclusions:**

The frequency of CT head usage in the studied patient population was high while the yield was low. This calls into question the usefulness of CT head in this population.

## Introduction

Hepatic encephalopathy (HE) is a clinical hallmark of end-stage liver disease (ESLD), characterized by recurrent episodes of confusion, disorientation, and altered mental status (AMS), which often result in frequent visits to the Emergency Department (ED).^1–4^ Despite its clinical significance, the presenting symptoms of HE are non-specific which can pose a diagnostic challenge for clinicians.^4,5^

Computed tomography (CT) of the head is often used as a diagnostic tool in patients presenting with AMS, including patients with suspected HE.^4,5^ However, the lack of HE-specific findings on CT scans calls into question its utility in the management of patients with suspected HE, especially in the setting of recurrent presentations.^6,7^

Presumably, one rationale for CT head is to exclude other causes of AMS, though supportive evidence is lacking. Current guideline from the American Association for the Study of Liver Diseases (AASLD) suggests the use of brain imaging for first-time presentation of HE to exclude other causes of neurologic dysfunction.^1^ However, this statement is only supported by a single case-control study demonstrating increased risk of intracranial haemorrhage (ICH) in patients with cirrhosis (adjusted odds ratio = 5.1, 95% confidence interval: 3.1–8.5).^8^ A subsequent review of 462 CT head for patients with cirrhosis demonstrated new ICH in 3% of the scans. Importantly, this study highlighted greater rates of ICH for patients with cirrhosis who presented with fall (5%), focal neurologic deficit (11.1%), and prior abnormal imaging (26.3%), as compared to those with solely AMS (0.34%).^9^ These results suggest a lack of utility for CT head for patients with cirrhosis without high-risk features. Similar findings have been documented in other published studies.^5,10,11^

The primary goals of this retrospective chart review were to assess (1) the frequency of CT head use in patients with known liver cirrhosis presenting to the ED with suspected HE and (2) whether these scans identified any significant intracranial pathologies. Our secondary goals were to (1) examine which patient factors may have contributed to the decision to order a CT head and (2) whether patients with CT scans had different clinical outcomes.

## Methods

We conducted a retrospective chart review of patients with known liver cirrhosis who were admitted to the hospital with either a first or recurrent episode of HE. This study was approved by the Research Ethics Board of the University of Alberta.

### Patient identification, inclusion, and exclusion

To assess the usefulness of CT head in our study population, we first identified patients with known cirrhosis who were hospitalized with signs and symptoms consistent with HE.

The charts of patients aged ≥18 who were discharged from the University of Alberta Hospital between January 1 2016—March 31, 2018 with the following primary ICD-10-CA diagnosis codes were identified for chart review: K70.1 (alcoholic hepatitis), K70.3 (alcoholic cirrhosis of liver), K70.4 (alcoholic hepatic failure), K71.1 (toxic liver disease with hepatic necrosis), K72.0 (acute and subacute hepatic failure), K72.1 (chronic hepatic failure), K72.9 (hepatic failure, unspecified), K74 (fibrosis and cirrhosis of liver), K74.0 hepatic fibrosis, K74.3 (primary biliary cirrhosis), K74.4 (secondary biliary cirrhosis), K74.5 (biliary cirrhosis, unspecified), and K74.6 (other and unspecified cirrhosis of liver).

The search codes included the ones that captured all patients, who were admitted with a diagnosis of cirrhosis. A trial search using these codes confirmed that they captured patients with cirrhosis including those with HE. To the best of our knowledge, these codes have not been validated previously in a separate study. The terms confusion and encephalopathy were not helpful search terms beyond the codes for cirrhosis. Codes related to viral hepatitis, including B15 (acute hepatitis A), B16 (acute hepatitis B), and B19 (unspecified viral hepatitis) were found not helpful in identifying patients with cirrhosis presenting with suspected HE in our preliminary search, thus were not included in our final search and data extraction.

Each chart represented one patient encounter. Patients who had multiple encounters during the study period had multiple charts extracted. All encounters were analyzed as independent events. The charts were manually reviewed by one author (D.Y., J.B., or M.A.) to confirm their eligibility. For this study, we included patients aged ≥ 18, with pre-existing diagnosis of cirrhosis, presenting with symptoms consistent with HE. Pre-existing diagnoses of cirrhosis were made based on documentation in the patient chart or prior imaging reports demonstrating liver cirrhosis. Symptoms of HE for this study include confusion, disorientation, inattention, behavioural changes, AMS, and altered level of consciousness (LOC).

As there was no absolute gold standard for diagnosing HE, inclusion for suspected HE was based on a combination of clinical symptoms, physical exam findings, and lab tests, including liver enzymes, INR, albumin, and ammonia levels. Patient’s progress notes and discharge summaries were also included in our chart review to ensure that HE was suspected at the time of admission to the hospital. We did not include patients with new diagnoses of cirrhosis because, without advanced knowledge of their pre-existing cirrhosis, ED providers may be treating them as undifferentiated delirium and search for alternative causes of their presentation.

Patients with other strong indications for CT head, such as a history of structural brain lesions, head trauma, new onset seizures, or focal neurologic deficits were excluded from this study. Patients presenting with other complications of cirrhosis, such as ascites and variceal bleeding, without evidence of HE, were not included. We did not document details on the number of excluded patients if they did not meet inclusion criteria.

### Clinical outcome and data collection

We tracked the frequency of CT head usage for patients meeting our inclusion and exclusion criteria and documented whether these scans discovered clinically significant findings. We defined clinically significant findings as any abnormal findings other than microangiopathic changes or expected generalized atrophy for the patient’s age. We made distinctions between CTs that were ordered in the ED and those ordered after admission to hospital.

To assess factors associated with the decision to order CT scans, multiple data were extracted including (1) patient characteristics—age, sex, cirrhosis aetiology, history of previous HE; (2) previous similar presentations, length of time between current and most recent presentation for HE, date of most recent head imaging; (3) patient presentation—presenting symptoms, vital signs, Glasglow Coma Scale (GCS) as documented by ED triage note, neurologic exam findings; (4) laboratory investigations—haemoglobin (g/L), platelet count (×10^9^/L), white blood cell count (×10^9^/L), international normalized ratio (INR), sodium (mmol/L), potassium (mmol/L), bicarbonate (mmol/L), creatinine (umol/L), urea (mmol/L), total bilirubin (umol/L), serum albumin (g/L), ammonia (umol/L), AST (U/L), ALT (U/L), ALP (U/L). Not all clinical and laboratory data were clearly documented in the charts. The GCS score was extracted as a variable if it was recorded in the chart at the time of admission, but no further details on this were collected.

The extracted data were analyzed to see whether there were differences between patients who received CT head and those who did not. For patient outcomes, we collected patient’s length of stay, ICU admission, and in-hospital death and compared the outcomes between patients with and without CT head. The costs of investigations were not assessed in this study.

### Statistical analysis

Comparisons of patient characteristics and clinical outcomes were made between those who received CT head in ED (CT head group) and those who did not (non-CT head group). Continuous variables were presented as mean values. Categorical variables were expressed as frequencies and proportions. A *T*-test was used to compare continuous data, while chi-square test was used for categorical data. Patients with >1 ED visits were analyzed as independent events, which simplifies the logistic regression analysis, avoiding the need to include random effects. The association between the performance of CT and inpatient mortality and length of stay was analyzed with logistic and ordinal regression, respectively. The confounding effect of the severity of liver disease was captured by adjusting for MELD-Na. Statistical significance was defined as a *P*-value < .05. Statistical analyses were performed using Microsoft Excel and R.

## Results

### Patient encounters

**Figure 1.**
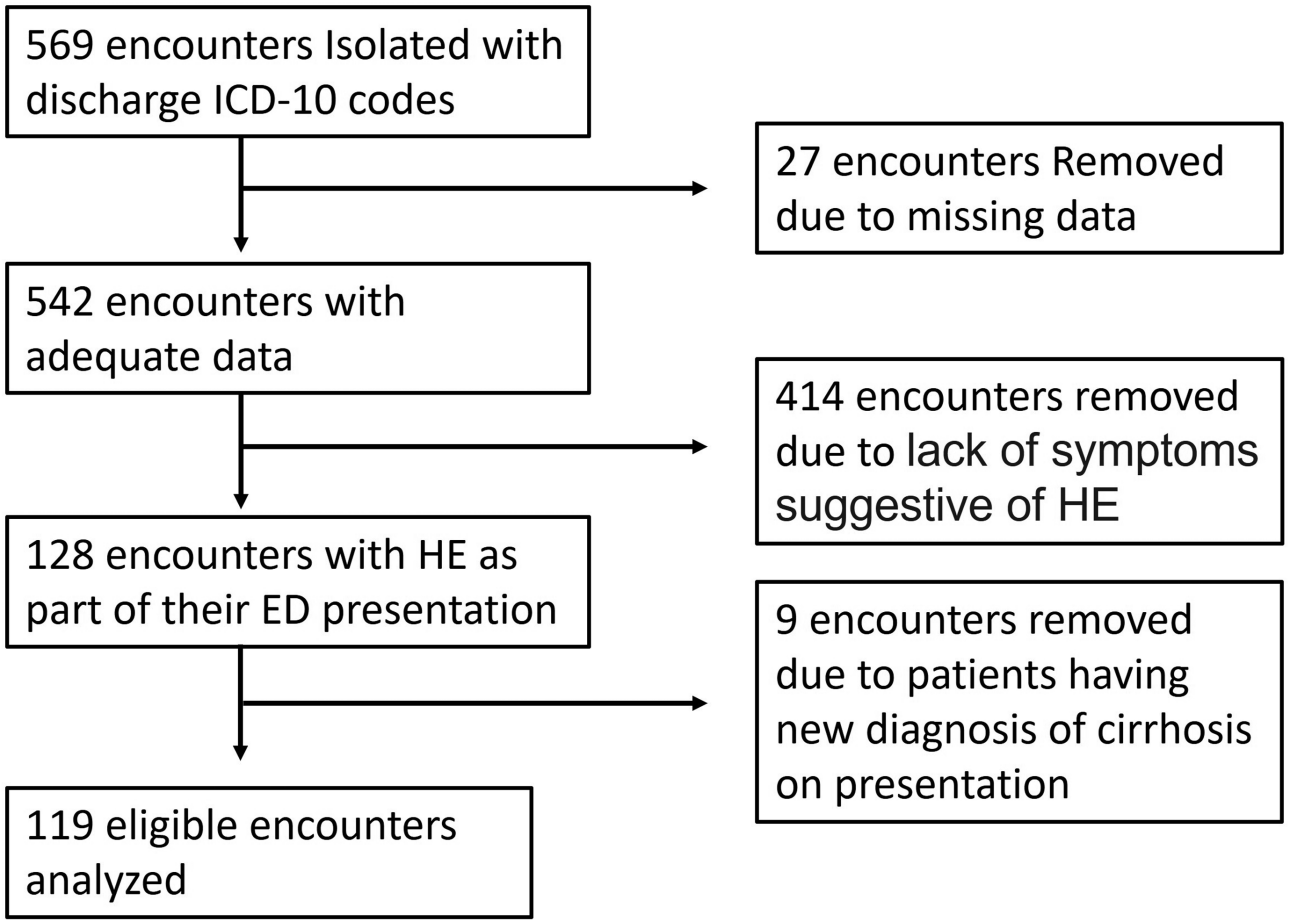
Patient identification. A total of 569 charts were isolated based on discharge ICD-10 codes. Charts with missing data, patients without HE on presentation, and patients without pre-existing diagnosis of cirrhosis were removed. This left 119 charts from 100 unique patients that met our inclusion and exclusion criteria.

A total of 569 encounters were identified based on discharge ICD-10-CA codes. Due to incomplete clinical data, 27 encounters were eliminated and due to the absence of HE symptoms at presentation, 414 contacts were eliminated. A further 9 encounters were removed due to lack of pre-existing cirrhosis diagnosis. In total 119 encounters from 100 unique patients met our inclusion criteria (Figure 1). The main presenting symptoms, as documented on the ED chart, were confusion, disorientation, and altered LOC. The frequency of patients having these symptoms did not differ between patients who received CT head and those who did not (Table 1).

**Table 1.** Patient characteristics of CT head and non-CT head groups.

Characteristics	CT head group	Non-CT head group	*P* value
Mean age (years) (±SD)	64 (±11.0)	58 (±11.8)	**.0025**^
Male *n* (%)	38 (55.9)	26 (51.0)	
Female *n* (%)	30 (44.1)	25 (49.0)	.59 ^*^
Alcohol-related cirrhosis *n* (%)	27 (39.7)	25 (49.2)	.31^*^
Days to previous HE presentation (±SD)^†^	73 (±93.1)	37 (±53.5)	**.027**^
Patients with prior CT scan within __ days of current presentation *n* (%)^‡^			
30 days	8 (16.0)	6 (17.6)	.84^*^
60 days	16 (32.0)	15 (44.1)	.083^*^
180 days	30 (60.0)	26 (76.4)	.14^*^
No CT scan within 1 year	19 (38.0)	6 (17.6)	**.045** ^*^
Mean GCS (±SD)	12.6 (±2.4)	13.2 (±1.8)	.090^
Presenting symptom *n* (%)			
Confusion	50 (73.5)	37 (72.5)	.90 ^*^
Disorientation	17 (25.0)	11 (21.6)	.66^*^
Decreased LOC	22 (32.4)	16 (31.4)	.91^*^
Other^§^	11 (16.2)	8 (15.7)	.91^*^

Abbreviations: GCS, Glasgow Coma Scale; LOC, level of consciousness.

^*^Chi-square test. ^*T*-test. Statistically significant values in bold.

^†^Only patients with encounters within one year of the current presentation (44 encounters from the CT head group and 34 encounters from the non-CT head group) were included in this analysis.

^‡^Fifty encounters from the CT head group and 34 encounters from the non-CT head group were included in this analysis due to lack of data on the remaining encounters.

^§^Other symptoms included weakness, lethargy, aggression, and other behavioural changes.

In our study design, we planned on excluding patients with strong indications for CT head such as those with a history of structural brain lesions, head trauma, new onset seizures, or focal neurologic deficits. However, we did not identify any patients meeting this exclusion criteria. Most ED charts did not contain detailed neurologic exam findings or explicitly stating a positive or negative history of trauma, structural brain lesion, seizures, etc.

### CT head utilization

Out of the 119 encounters, 68 (57%) had CT head performed on presentation. This constitutes the CT head group; 97% of scans (66/68) were ordered by ED physicians and 3% (2/68) were ordered by the inpatient gastroenterology (GI) service. The mean time from ED registration to CT completion was 4.45 ± 3.64 h. None of the CTs ordered on presentation demonstrated any clinically significant findings. Of the 68 encounters in the CT head group, 8 had repeat CT after admission. These inpatient CT scans did not yield any clinically significant findings either.

In the non-CT head group, 4 of 51 encounters had CT head done during their hospitalization. One CT head showed a new ICH. This scan was ordered on day 7 of admission following new-onset seizures. No neurologic deficit was documented on this patient’s ED chart at the time of presentation.

### Patient factors associated with CT usage in ED

When comparing the CT head group to the non-CT head group, patients in the CT head group were older (64 vs 58, *P* = .0025). The CT group had a longer length of time between their current and previous ED visit for HE (73 days vs 37 days, *P* = .027). More patients within the CT head group have not had a prior CT head within 1 year of ED presentation (38.0% vs 17.6%, *P* = .045) (Table 1). When comparing their liver function, the CT head group had lower INR (1.5 vs 1.7, *P* = .0025), lower total bilirubin (62.5 vs 100.3 umol/L, *P* = .012), and lower MELD score (15.1 vs 18.9, *P* = .003) (Table 2).

**Table 2.** Comparison of laboratory parameters of CT head and non-CT head groups.

Laboratory parameter	CT head group	Non-CT head group	*P* value^
Hb (g/L) (±SD)	110.2 (+21.3)	96.3 (±24.1)	**.00064**
Plt (× 10^9^) (±SD)	100.8 (+48.7)	129.6 (+72.2)	**.0056**
Creatinine (umol/L) (±SD)	113.1 (+64.8)	139.2 (+121.4)	.07
Na (mmol/L) (±SD)	135.6 (±5.0)	134.7 (+5.5)	.17
INR (±SD)	1.5 (±0.4)	1.7 (±0.6)	**.0025**
ALT (U/L) (±SD)	37.6 (+19.9)	59.3 (+34.7)	**.015**
AST (U/L) (±SD)	70.1 (+56.3)	96.7 (+63.5)	**.043**
Total bilirubin (umol/L) (±SD)	62.5 (+62.2)	100.3 (+114.0)	**.012**
Albumin (g/L) (±SD)	30.4 (±5.1)	28.7 (±7.3)	.105
Ammonia (umol/L) (±SD)	106.8 (+61.0)	91.9 (+44.5)	.086
MELD (±SD)	15.1 (+6.0)	18.9 (9.1)	**.003**

^*T*-test.

Sex, GCS, creatinine, and percentage of patients with alcohol as a cause of cirrhosis did not differ between the 2 groups. Serum ammonia levels were tested for 105/119 (88%) of patients. Average ammonia levels did not differ significantly between the 2 groups (107 vs 92 umol/L, *P* = .086) (Table 2). The frequency of CT scans between patients with normal and elevated serum ammonia levels (defined as >50 umol/L) did not differ significantly. CT scans were performed on 50% (8 out of 16) of patients with normal ammonia levels and 57.3% (51 out of 89) of patients with increased ammonia levels (*P* = .29)

### Outcomes

Patients who received CT head had a shorter length of hospitalization (11 vs 17 days, *P* = .024) and lower in-hospital mortality (1.5 vs 28.8%, *P* = .000023). When adjusted for MELD-Na, these associations were completely abrogated (*P* = .10 and *P* = .73, respectively) indicating that they were confounded by the severity of liver disease. There was a trend towards more ICU admission for the non-CT head group, but the difference was not statistically different (13.3% vs 23.5%, *P* = .051).

## Discussion

In this review of 119 encounters of patients presenting with suspected HE, the majority (68/119) received CT head in ED. None of these CT scans had significant findings. The average time from ED registration to completion of CT scan was only 4.45 ± 3.64 hours. This means the decision to order these CTs was made quickly after the initial assessment.

The only CT scan with a clinically significant finding was performed on a patient who had a seizure on day 7 of hospital admission. This patient was admitted for HE and showed clinical improvement until the occurrence of a seizure. As there was no documentation of localizing neurological findings at the ED presentation, this seizure event was a new neurological symptom. In this case, CT head was the appropriate diagnostic tool at the time of the seizure but not indicated at the time of admission.

Overall, our results indicated CT head has a low yield in patients with known cirrhosis presenting with suspected HE in the absence of focal neurologic deficits, documented history of falls/trauma, or prior abnormal brain imaging. In the absence of these high-risk features, starting a therapeutic trial of HE treatments while ruling out other contributing causes for HE, such as dehydration and infection, is a reasonable approach. Immediately ordering a CT head upon patient presentation to ED is unlikely necessary. The CT head can be considered if the patient does not improve with empiric treatment. Through our observation, we suspect that the high frequency of ordering CT heads was explained by the fact that the test is considered part of a general workup of patients presenting with confusion, without taking into consideration the clinical context of their cirrhosis. Our hospital does not have a specific algorithm for diagnostic work-up of patients with cirrhosis presenting with confusion. Based on our findings, as well as prior publications, one may postulate that a more judicious approach to CT head usage would be to reserve the test for those with high-risk features such as history of falls or head trauma, focal neurologic deficits, and prior abnormal CT head. This may lead to cost savings and decrease unnecessary radiation exposure. The costs and harms of unnecessary investigations are important but were not assessed in this study.

Patient factors associated with the decision to order CT included older age, lower MELD score, lack of previous head CT within 1 year of current presentation, and longer length of time between the current and previous ED visits. These patient factors likely reflect the decision-making process by ED physicians. The tendency to order CTs for older patients may be due to higher concern for intracranial pathologies in this population. In patients with a lower MELD score, there was an increased tendency to order CT head. This may be due to a higher level of concern for alternate causes of AMS given their more preserved liver function. Patients who had a more recent ED visit for decompensated cirrhosis had a lower probability of having a CT head. More recent ED visits may have reassured the ED physicians that the patient’s current presentation was in keeping with HE as opposed to an alternative diagnosis. In our cohort, patients with CT head had lower platelet count (100.8 vs 129.6, *P* = .0056) and lower INR (1.5 vs 1.7, *P* = .0025) as compared to those without CT head. It is unclear whether these 2 haemostatic indicators directly influenced the decision for CT head, but prior studies have demonstrated a lack of correlation between INR and platelet count and rate of ICH.^9^ It is important to highlight that existing evidence does not support using INR and platelet count to assess the pre-test probability of ICH in patients with cirrhosis.

Although serum ammonia level has poor diagnostic accuracy and limited clinical utility in the management of HE, this test was ordered frequently.^12^ However, the ammonia level did not differ significantly between the CT head and non-CT head group. Patients with elevated ammonia received CT scans at the same frequency as those with normal ammonia. Given the short time between the patients’ ED registration and CT completion, it is possible that that decision for CT head was made before the ammonia level was reported, and if so, ammonia levels would not have impacted the decision for CT.

The length of hospitalization and in-hospital mortality was significantly higher for the non-CT group. However, since none of the CTs ordered in ED had any significant findings, it is doubtful that ordering the scans contributed to the differences in patient outcomes. Rather, the differences in outcome were more likely related to the increased severity of liver disease in patients who did not receive CT.

There are several limitations to this study. First, our method of patient identification had the potential for selection and/or ascertainment bias. Potential study patients were identified based on their primary ICD-10-CA diagnostic codes. These ICD-10-CA codes are related to liver disease or complications of cirrhosis. The included search codes captured all patients who were admitted with a diagnosis of Cirrhosis. The terms confusion and encephalopathy were not helpful search terms beyond the codes for cirrhosis. It is possible that patients with cirrhosis who presented with altered mental status from non-HE causes, such as intracranial hemorrhage, may carry a discharge diagnosis code that did not include a cirrhosis diagnosis. Such patients would then have been missed during our patient identification process. This could potentially lead to the underestimation of the usefulness of the CT head. We do not know how many cirrhosis patients were excluded because of overt neurological findings. However, the number of these types of patients is likely low.

Second, the retrospective design predisposed the study to misclassification bias of study subjects. This was mitigated by using broad ICD-10-CA codes to capture potential charts followed by a manual review to ensure they met inclusion criteria. Third, some data of interest were missing from many patient records. These included the presence of asterixis, presence of ascites, neurologic exam findings, Child-Pugh score, history of non-compliance with treatment, grading of patient’s HE, and previous HE treatment. Medication lists in ED charts were at times, incomplete, thus it was not possible to reliably determine whether the patient was on anticoagulation prior to the ED visit. The role of anticoagulation in the decision for CT head could not be assessed. Furthermore, this was a single-centre study in a quaternary centre only examining patients presenting to the ED, so the results of this study may not be generalizable across all healthcare settings including hospital inpatients with HE onset after hospitalization. An additional limitation is our small sample size, in comparison to a similar study.^9^ Lastly, information as to why CT was not performed in the other 43% of patients was not collected. This would be difficult to do in a retrospective study.

Despite these limitations, our study does have important implications for the management of HE. This study has demonstrated that the yield of CT head is low in patients with known cirrhosis presenting with altered mental status without focal neurologic deficits. This suggests clinicians should be more prudent when considering CTs in this population. It may be appropriate to empirically treat these patients for HE and to assess for response before ordering a CT head.

## Conclusion

Despite the low yield, the frequency of ordering CT scans of the head is high in cirrhosis patients presenting with symptoms of HE to the ED. CT head were less likely to be ordered for patients with worse liver function and those with shorter time intervals between current and previous HE presentations. Restricting CT head to patients with known high-risk features, such as the history of falls, focal neurologic deficits, and prior abnormal head imaging, will likely decrease the number of low-yield CT scans.

## Supplementary Material

gwae022_suppl_Supplementary_Checklist

gwae022_suppl_Supplementary_Material

## Data Availability

The data underlying this article will be shared on reasonable request to the corresponding author.
